# Translation and Cultural Adaptation into Portuguese of the Quality of Dying and Death Scale for Family Members of Patients in Intensive Care Units

**DOI:** 10.3390/ijerph19063614

**Published:** 2022-03-18

**Authors:** Silmara Meneguin, Cariston Rodrigo Benichel, José Fausto Morais, Cesar de Oliveira

**Affiliations:** 1 Department of Nursing, Botucatu Medical School, Paulista State University, Sao Paulo 18618-687, Brazil; c.benichel@hotmail.com; 2 Faculty of Mathematics, Universidade Federal de Uberlândia, Santa Monica 38408-100, Brazil; jfmorais.ufu@hotmail.com; 3 Department of Epidemiology & Public Health, University College London, London WC1E 7HB, UK; c.oliveira@ucl.ac.uk

**Keywords:** death, quality of dying and death scale, translation, cross-cultural adaptation, validation studies, intensive care

## Abstract

The translation and cultural adaptation of the Quality of Dying and Death in Brazil may provide a reliable and reproducible scale for collecting and analyzing data on the process of dying and death, given the absence of Brazilian studies that have produced or used scales in this topic. The purpose of this study was to perform the translation and cultural adaptation of the Quality of Dying and Death (QODD 3.2a) scale for intensive care patients’ relatives into Portuguese (Brazil). This methodological study was carried out in a public university of the São Paulo State University (UNESP) medical school, São Paulo, Brazil, in three stages: translation and back-translation by two native-speaking independent professionals, analysis by a committee of specialists, and a pre-test phase. The final version was created by seven experts after making semantic, idiomatic, and cultural changes to 16 items. The results indicated a satisfactory content validation index (CVI ≥ 0.80). This version was applied on 32 relatives of patients who were hospitalized in a public hospital in the interior of São Paulo. No item was excluded from the instrument. The content and face validity were achieved to a satisfactory standard, in addition to reaching the minimum parameters recommended in the literature. The Portuguese version of QODD 3.2a for relatives of deceased patients in intensive care is appropriate and culturally adapted for use in Brazil.

## 1. Background

The moment of death underwent significant changes throughout the industrialization and urbanization processes in the 19th century. This grieving scenario gradually distanced itself from homes and was transported to hospital beds as Western religiosity declined and knowledge about disease treatment increased [1].

The reframing and provision of care sparked interest in coping with death, as well as debates concerning the quality of dying [2]. At the same time, there were considerations of potentially avoidable anguish and suffering, as well as the balance between desires, ethical and cultural standards, and individual needs for preparation and coping [2,3,4,5].

Various methods have been used to collect this data and reduce the subjectivities associated with death. However, a more in-depth examination of the experiences and perceptions of family members of deceased patients has proven to be one of the best ways to identify elements capable of improving the quality of care provided to a loved one in life [2,6].

In 2001, North American researchers from the University of Washington, Seattle, USA, proposed measuring the quality of death and dying, as well as evaluating a person’s and their family members’ perceptions of death, through the development of an instrument called the Quality of Dying and Death (QODD) [6,7]. It was created through a review of existing instruments, interviews with patients to define domains around death, qualitative research with patients to explore states of good or bad death, and the definition of the desired instrumental profile [8].

The authors then conducted a validation study with 147 family members of patients who died in Missoula-Montana between 1996 and 1997, drawn from a database of 1082 deaths in this municipality [6]. The initial instrument consisted of 31 questions divided into six domains, with a total score ranging from 0–100, with higher scores indicating higher quality. During the initial validation process, these scores averaged 67.36 (15.06), and Cronbach’s alpha for the instrument’s total score was 0.86 [6,9]. Following that, in a 2010 study, a confirmatory factor analysis and exploratory study were developed [10].

It is worth noting that the QODD does not result in a static definition of death and the dying process, but rather aims to estimate measurable aspects of the end-of-life experiences of patients, family members and caregivers [2,7]. It can be stated that the use of this instrument aids in the decision-making process in the face of death by providing a concrete assessment of the subjective concepts associated with it [2,4,5,7,11]. The Spanish, German, Chinese and Persian versions of the QODD have already been culturally adapted and validated [12,13,14,15,16].

The QODD version 3.2a was created after nine items were eliminated as being inappropriate for the intensive care setting, resulting in an instrument with 22 items applicable in the Intensive Care Unit (ICU). The same classification was maintained (with scale from zero to ten to represent a “terrible experience” or “almost perfect”, respectively). In addition to these 22 items, three others were added to improve overall quality of life while maintaining the original instrument’s reliability [17]. The QODD version 3.2a has 25 items. Twenty-two of the items assess aspects related to the experience of respondents during their loved one’s last days. Three items assess the quality of care delivered by the health team and the quality of dying. The instrument is divided into six domains as follows: (1) symptoms and personal control (pain under control, control over what was going on around him/her, ability to feed himself/herself, breathing comfortably); (2) preparation for death (felt at peace with dying, unafraid of dying, bad feelings spoken out, said goodbye to loved ones, health care costs covered, spiritual advisor visits, spiritual service or ceremony before death, had funeral arrangements in order); (3) moment of death (was anyone present at the moment of death?, state before death i.e., sleeping, awake or unconscious); (4) family (spent time with family, spent time alone); (5) treatment preferences (discussed end of life wishes with physician, experience of mechanical ventilation as an aspect of dying, experience of dialysis); and (6) whole person concerns (ability to laugh and smile, maintained dignity and self-respect, was touched and hugged by loved ones) [2,6].

Given the absence of Brazilian studies that produced or used scales in the context presented, the translation and cultural adaptation of QODD 3.2a in Brazil may provide a reliable and reproducible scale for collecting and analysing data on the process of death and dying, as well as the respect for multiple experiences in the face of the finitude of life. Therefore, this study aimed to perform the translation and cultural adaptation of the QODD 3.2a for intensive care patients’ relatives into Portuguese (Brazil).

## 2. Methods

### Study Design and Survey Development

**Figure 1:**
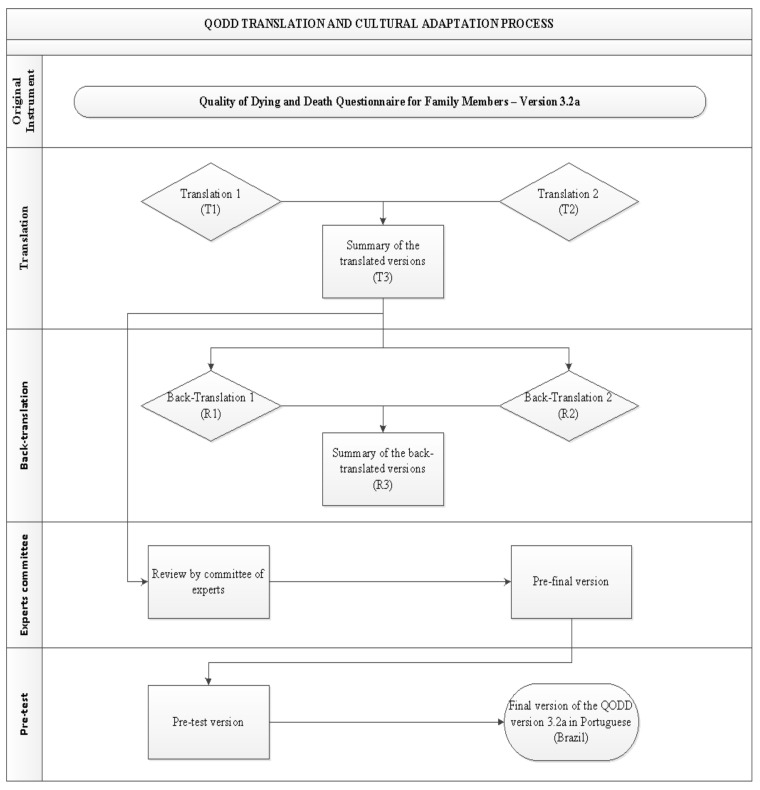
Flowchart of the QODD translation and cultural adaptation process into Portuguese, Botucatu, SP, Brazil, 2021.

This is a methodological study of translation and cross-cultural adaptation of QODD 3.2a into Portuguese spoken in Brazil, carried out from January 2020 to December 2020, according to international recommendations, namely [18]:

1. Translation: It was carried out by two bilingual translators (Portuguese-English) who were native to Brazil and had no prior knowledge of the questionnaire. T1 and T2 were the new versions of the scale, and from these, the summary, T3, was obtained;
2. Back-translation: This step was necessary to verify that the translated version reflected the same content as the original version, in addition to any inconsistencies [18]. As a result, version T3 was back-translated into English by two other bilingual natives who had no prior knowledge of the questionnaire, resulting in two back-translated versions named R1 and R2, which were later summarized into a single version named R3;
3. Committee of Experts: The final translated version was evaluated by a Committee of Experts to obtain cross-cultural equivalence. For the Expert Committee, seven academics who had clinical experience and publications in the areas of intensive care, palliative care and protocol management were selected. They were selected, based on their scientific knowledge, from a national academic database called Platform Lattes. It is a database that contains all researchers’ curriculum details, their research groups, and areas of expertise from all higher education Brazilian institutions. Next, an invitation email was sent to each of the selected experts including a term of agreement, a copy of the translated instrument i.e., version T3 and a form with instructions as well as the study objectives and how to conduct their analysis/assessment. They used a Likert scale with a score from 1 to 4: 1 (non-representative item), 2 (item needs major revision to be representative), 3 (item needs minor revision to be representative) and 4 (relevant or representative item). The Committee also evaluated the instrument in three categories: semantics, idiomatic and cultural. A Committee of Experts evaluated Version T3 to determine cross-cultural equivalence. The content validity index (CVI) was calculated, considering notes 3 and 4, added and divided by the total number of responses, inferring adequacy of items with CVI ≥ 0.80 [19,20,21].
  The committee had 15 days to complete their preliminary analyses, and, after their feedback, a synthesis of their individual recommendations was carried out. A face-to-face meeting with the experts was not possible due to COVID-19 restrictions at the time.
4. Pre-test phase: This phase was conducted with family members who agreed to participate in the study and responded to the pilot version of the instrument. Invitations to participate were made via telephone, followed the electronic access to QODD at the address. First, the participating institutions provided the list of patients who have died in their intensive care units in the last month. Next, we contacted via telephone potential respondents who were aged 18 or older and were closely involved in the dying process of their loved one in the period between 30 and 90 days after their death and consented to participated in the present study. Those individuals who reported not having emotional capacity or were not located were excluded. The sample was defined based on previous literature [18] that recommends between 30 to 40 participants for this stage which seeks to assess whether all items of the instrument were adequate and understandable to the target population.
  In addition to the items included in the pre-test version, participants answered a few additional questions: How many stars they would give the questionnaire, with 1 (one star) being the worst possible rating and 5 (five) stars being the best possible rating; and what may have made it difficult to understand while filling out. When the items had a minimum agreement of 75% in the positive responses, they were considered validated. Minor agreement items were thought to be subject to change [22]. Figure 1 depicts all stages of the translation and cultural adaptation process.
  This study obtained authorization from the authors of the original instrument, as well as, the consent of the Research Ethics Committee, according to the consubstantiated opinion number 2,772,325; and was structured according to guideline recommendations Standards for Quality Improvement Reporting Excellence (SQUIRE 2.0) [23], and by COnsensus-based Standards for the selection of health Measurement INstruments (COSMIN) [24].

## 3. Results

The process of translation and cultural adaptation of the QODD to the Portuguese language spoken in Brazil was carried out as proposed in the method of this study, obtaining the summary in a single version called T3; this in turn was subjected to the back-translation process into English, and the summary in a single version called R3.

A Committee of Experts evaluated Version T3 to determine cross-cultural equivalence. Seven judges were chosen for their convenience through non-probabilistic sampling by analyzing their curriculum and scientific knowledge. They all had clinical experience and publications in the areas of intensive care, palliative care, and protocol management, with the majority being women (71.4%), two doctors (adult intensivist and palliative care), one social worker, and four nurses. Of these, 14.3% had a PhD degree, and all had an average of 22 (SD ± 14) years of training. 80% reported having experience in the field of psychometry.

Each of the 25 items that make up QODD 3.2a, as well as the 14 sociodemographic characteristics, were evaluated in the semantic, idiomatic, and cultural categories. They had the following average score related to CVI: Semantics = 0.99; Idiomatic = 0.98; Cultural = 0.99.

The global average for CVI was 0.99, indicating an acceptable value and adequate representation (≥0.80). Ninety-four percent of the items in the semantic and idiomatic categories and 85% of the cultural category were assessed with CVI = 1.0. Items that did not receive the maximum CVI score received a value of 0.86. These, as well as the other items that have been modified, are underlined and highlighted in bold in Table 1.

Considerations were made for 11 items: 5a, 14a, 15a, 16a, 17a, 18a, 19a, 20a, 21a, 25a and 1–22b. The main suggestions included adjustments of verbal agreement, change of synonyms and restructuring of the question to favor a better understanding. The items and respective categories that reached the CVI = 0.86 were: 14, 16 and 25 (cultural) and 17 and 19 (cultural, semantic and idiomatic). The experts suggested adjustments to the verbal agreement of the presentation text and reformulation of the wording of the guidance that precedes item 11a, for a better reference to items 11a–21a.

Only questions 17a and 19a received CVI = 0.86 in all categories (semantic, idiomatic, and cultural), encompassing the use of ventilatory therapies and funerary measures by the deceased while they were still alive. Item 14a was the most altered, and from the perspective of the specialists, it resulted mainly from the cultural dimension (CVI = 0.86).

Since no item in the QODD was considered inappropriate, there was no exclusion of the questions the original instrument or its respective pre-test version (T3). The pre-test version was proposed for 33 family members who experienced the death process of patients assisted in the ICU. Of these, 32 (97%) agreed to participate. The main cause associated with refusals was the grieving process.

The average age of the family participants was 47 years old (SD 18), with 17 of them being female (62%), Brazilian (100%), Caucasian (81%), and mostly having completed a higher education (28%) or a high school diploma (16%). The condition of being a child was the most prevalent degree of kinship (66%). They reported having lived with the deceased loved one (69%) and meeting them on average for 41 years (SD ± 15). As for the hospital stay, the deceased ones spent, on average, 33 (SD ± 27) hospitalized days, 20 (SD ± 17) of them in the ICU.

The average time to answer the scale was 19 min (SD ± 13), and the majority (91%) understood and finished the entire questionnaire. Of the three (9%) who did not fully understand it, they made mention of items 1–11b “How would you rate this experience of the process of dying of your loved one?”; and 12a “Have all the costs of caring for your loved one been paid?”. One participant suggested adding a topic about the experience with the visits and interaction with the health team, and another reported feeling pain when reliving such experiences.

In summary, QODD 3.2a remained similar to the original instrument, with the same number of items proposed in the translated and culturally adapted version for Portuguese (Brazil).

## 4. Discussion

The validation of pre-existing instruments, in addition to being less expensive and less complex than the new development of instruments, indicates pertinent action to assess population patterns. In this case, we start from primary experiences, whose constructs remain in the same perspective of analysis [25].

Some of the key features to develop a research instrument to be used in the health context include good construct and item organization to facilitate the comprehension of its context combined with its reliability. This way, an instrument that presents such characteristics from its formulation stage is a good option for cultural translation and validation into other languages such as the Amsterdam Preoperative Anxiety and Information Scale that has been translated and validate into German, French, Malay and Japanese [26].

The current study followed the recommendations presenting a method similar to that of other comparable research, including an average contingent of two native translators at each stage of translation and back-translation and an average of at least five professionals for the composition of the Committee of Experts [12,13,14,15,16,25,27,28], to ensure greater reliability and credibility [16]. This step is described as critical for professionals’ inferences in the literature. It provides subsidies and adds significant value to the instrument’s understanding [29]. The Committees of Experts of other cultural adaptation studies of the QODD were composed of 6 to 12 professionals, including clinicians, psychologists, nurses and social workers [12,13,14,15,16].

Among the considerations made by the specialists in the present study, one involved the question 12a: “Have all the costs of caring for your loved one been paid?” Considering that the largest proportion of the Brazilian population are users of the Unified Health System (SUS), the relevance of maintaining it in the questionnaire was questioned. However, its maintenance was chosen, since it presented CVI = 1.0 and because the study indistinctly envisions the qualification of the dying and death process, regardless of the insertion in the public or private care setting.

Additionally, the instrument points out guidelines on how to proceed to return it by post if the QODD has been answered in the printed version: “Please, find attached an envelope with a seal […]”. One of the experts (E5) suggested the need to reformulate and simplifying it as follows: “[…] after completing the questionnaire, you must put it in the envelope that accompanies it and post it at the nearest post office to your residence […]”.

It is worth highlighting that each individual experiences the process of dying in a unique way. The discussions around death can be complex and subjective, and the use of instruments to understand the perceptions of those involved has improved interventions towards better care.

The second section of the QODD included questions on sociodemographic characteristics and assessment of the instrument. The Committee of Experts suggested changes to the following items of this section: 1, 2, 7, 8, 10, 11, 12 and 14. Items 1 and 2 underwent changes in the wording format. In its original version, item 7 relates to ethnicity: “Hispanic and non-Hispanic” ethnicity.

The translation of the original version (T3) changed the answers to “Brazilian and non-Brazilian” and, to adapt the context of the question, the experts even suggested changing the term “ethnicity” to “nationality”. The item 8 asked about race, but the terms of the original instrument are relevant to the sociodemographic context common to Nordic regions. This item was changed to: “white, black/Afro-American, yellow/Asian, brown and indigenous”. The Committee of Experts suggested changing the title of the question “What is your race?” to “What is your ethnicity?”. The other items (10, 11, 12 and 14) underwent only adjustments of temporal agreement of the verb and change of synonyms.

Considering such cultural differences and distinctions between the languages allowed the instrument to be adapted to the new reality to be applied. Other study have also mentioned the presence of words or expressions that have no equivalent terms in Portuguese should be translated contextually [28].

In the pilot phase of the present study, the Portuguese version of the QODD was very comprehensive and well received by 91% of the respondents. Our instrument was answered by all respondents in contrast to the multicentre Dutch study of the QODD that was administered to 100 family members of patients who died in three hospitals in Holland. In the Dutch study many items were not answered due to the moderate level of difficulty to complete their instrument. According to the authors, these findings highlighted not only the poor capacity of the Dutch instrument to identify a question as irrelevant or unclear but also the fact that the experiences reported may not be related only to differences in quality of care at end of life but to differences in expectations towards the critical care delivered. This may have contributed to different interpretations among respondents [4].

Our main findings showed that the adapted version of the instrument was well adapted to the Brazilian reality, in view of its content validity coefficient. Similar data were verified in another study which contextualized palliative needs in a translated and validated instrument with CVI = 0.94 [30]. In this sense, content validation allowed for the verification of how many of the included items corresponded to the theoretical construction underlying the instrument, being representative and relevant in covering the phenomenon in question [27]. It is not unusual during the translation and cultural adaptation process that some items are eliminated or modified. In previous studies of translation and cultural adaptation of the QODD into other languages, items no longer relevant to a particular social context, questions that are not applicable [12] and those with similar meaning were removed [17]. However, this was deemed unnecessary in the present study. The content validation index values of the present study were similar to other cultural adaptation and translation studies into other languages, with a small variance between 0.8 and 0.96 [13,15].

Our pre-test phase was conducted with 33 family members who were closely involved in their loved one’s dying process in an intensive care unit. Most of the studies using the QODD scale followed the same criteria with samples varying between 6 and 32 respondents [12,13,14,15,16].

However, the selection of an instrument developed in a different language, context and culture than the one in which it will be used is only the first step in a process necessary to make it reliable and capable of being correctly applied in another reality.

## 5. Limitations

The impossibility of the family members to experience the process of dying of their loved ones in conditions of restrictions or isolation were verified, which would influence in the perception of some of the items of QODD 3.2a.

In cases wherein the patients can’t express their feelings or desires the perceptions of family members could assume a certain subjectivity or imprecision.

## 6. Conclusions

The Portuguese version of QODD 3.2a for intensive care patients’ relatives was considered adequate and culturally adapted for use in Brazil. As a result, the instrument is ready for the next step, which is to evaluate its psychometric properties.

In view of the lack of studies involving intensive care patients’ relatives the translation and cross-cultural adaptation of this instrument to Brazilian Portuguese will allow health services to review their current approaches resulting in improvements in the care provided to family members of intensive care patients in Brazil.

Indeed, this instrument will allow for research to be conducted with family members of intensive care patients and, ultimately, and help to evaluate the quality of assistance provided by health professionals.

## Figures and Tables

**Table 1:** Items of the QODD version translated into Portuguese (Brazil) that had a content validation index = 0.86 and/or that underwent changes through inferences from the Experts Committee, Botucatu, SP, Brazil, 2021.

Item	Version	Text	IVC *		
			Semantics	Idiomatic	Cultural
**Section I—Quality of the Dying and Death Process**					
5a	Original	How often did your loved one appear to feel at peace with dying? (Circle one number).			
	Translation	How often did your loved one seem to feel at peace with death? (Circle a number).			
	Committee Outcome	How often did your loved one seem to feel at peace during the process of dying? (Circle a number).	1.00	1.00	1.00
Guidance	Original	The following questions are answered with either a “Yes” or “No” based on whether your loved one did certain activities. Please rate the quality of that aspect of the dying experience. Again, we are asking you to focus on your loved one’s last several days.			
	Translation	The following questions should be answered with “Yes” or “No”, based on whether your loved one has performed certain activities or not. Please rate the quality of that aspect of the dying experience. Again, we ask that you focus on the last days of your loved one.			
	Committee Outcome	The following questions should be answered with “Yes” or “No”, based on whether your loved one has performed or experienced any of the activities, procedures or situations listed in questions 11 through 21. Please rate the quality of that aspect of the dying process experience. Again, we ask that you focus on the last days of your loved one.	1.00	1.00	1.00
14a	Original	Did your loved one clear up any bad feelings with others? (Circle one number).			
	Translation	Has your loved one cleared up bad feelings with other people? (Circle a number).			
	Committee Outcome	Has your loved one resolved any disagreements with someone or exposed their negative feelings to others? (Circle a number).	1.00	1.00	0.86
15a	Original	Did your loved one have one or more visits from a religious or spiritual advisor? (Circle one number).			
	Translation	Has your loved one received one or more visits from a religious or spiritual counselor? (Circle a number).			
	Committee Outcome	Has your loved one received one or more visits from a religious or spiritual leader? (Circle a number).	1.00	1.00	1.00
16a	Original	Did your loved one have a spiritual service or ceremony before his/her death? (Circle one number).			
	Translation	Did your loved one have a spiritual service or ceremony before they died? (Circle a number).			
	Committee Outcome	Did your loved one receive any religious or ritual visits before they died? (Circle a number).	1.00	1.00	0.86
17a	Original	Did your loved one receive a mechanical ventilator (respirator) to breathe for him/her? (Circle one number).			
	Translation	Has your loved one received a mechanical ventilator (respirator) to breathe for him? (Circle a number).			
	Committee Outcome	Was your loved one under artificial respiration, that is, was he placed on mechanical ventilation (respirator) to breathe for him? (Circle a number).	0.86	0.86	0.86
18a	Original	Did your loved one receive dialysis for his/her kidneys? (Circle one number).			
	Translation	Has your loved one received kidney dialysis? (Circle a number).			
	Committee Outcome	Did your loved one undergo kidney dialysis? (Circle a number).	1.00	1.00	1.00
19a	Original	Did your loved one have his or her funeral arrangements in order prior to death? (Circle one number).			
	Translation	Did your loved one have funeral arrangements in place prior to his death? (Circle a number).			
	Committee Outcome	Were your loved one’s funeral arrangements organized before his death? (Circle a number).	0.86	0.86	0.86
20a	Original	Did your loved one discuss his or her wishes for end-of-life care with his/her doctor—for example, resuscitation or intensive care? (Circle one number).			
	Translation	Your loved one discussed your wishes for care at the end of life with his doctor, for example, resuscitation or intensive care? (Circle a number).			
	Committee Outcome	Your loved one discussed his wishes with his doctor about how he would like to be cared for at the end of his life, for example, resuscitation or intensive care? (Circle a number).	1.00	1.00	1.00
21a	Original	Was anyone present at the moment of your loved one’s death? (Circle one number).			
	Translation	Was anyone present at the moment of the death of your loved one? (Circle a number).			
	Committee Outcome	Some family members were present at the moment of the death of your loved one? (Circle a number).	1.00	1.00	1.00
1–22b	Original	How would you rate this aspect of your loved one’s death? (Circle one number).			
	Translation	How would you rate this aspect of your loved one’s death? (Circle a number).			
	Committee Outcome	How would you rate this experience of the death of your loved one? (Circle a number).	1.00	1.00	1.00
25	Original	Rate the care your loved one received from his or her doctor during the last several days of his or her life while in the ICU. (Circle the number).			
	Translation	Rate the care your loved one received from his doctor in the last days of his life while he was in the ICU. (Circle a number).			
	Committee Outcome	Rate the care that your loved one received from his doctor (assistant or intensive care) in the last days of his life while he was in the ICU. (Circle a number).	1.00	1.00	0.86
Section II—Sociodemographic characterization					
1	Original	When were you born? (Please write the year).			
	Translation	When were you born? (Please write the year).			
	Committee Outcome	In what year were you born? (Please write the year).	1.00	1.00	0.86
2	Original	When was your loved one born? (Please write the year).			
	Translation	When was your loved one born? (Please write the year).			
	Committee Outcome	In what year was your loved one born? (Please write the year).	1.00	1.00	1.00
7	Original	What is your ethnicity? (Circle one number). 1. Hispanic or 2. Non-Hispanic.			
	Translation	What is your ethnicity? (Circle a number). 1. Brazilian or 2. Non-Brazilian.			
	Committee result	What’s your nationality? (Circle a number). 1. Brazilian or 2 Non-Brazilian.	1.00	1.00	1.00
8	Original	What is your race? (Circle all that apply). 1. White 2. Black/African American 3. Asian 4. Pacific Islander 5. Native American or Alaskan 6. Native 7. Other (please specify)			
	Translation	What is your race? (Circle all that fit). 1. White 2. Black/African American 3. Yellow/Asian 4. Brown 5. Indigenous 6. Other (please specify)			
	Committee Outcome	What is your ethnicity? (Circle all that fit). 1. White 2. Black/African American 3. Yellow/Asian 4. Brown 5. Indigenous 6. Other (please specify)	1.00	1.00	1.00
10	Original	How are you related to your loved one? (Circle one number).			
	Translation	What is your relationship with your loved one? (Circle a number).			
	Committee Outcome	What is the degree of kinship with your loved one? (Circle a number).	1.00	1.00	1.00
11	Original	Did you live with your loved one? (Circle one number).			
	Translation	Did you live or lived with your loved one? (Circle a number).			
	Committee Outcome	Did you live or lived with your loved one? (Circle a number).	0.86	0.86	0.86
12	Original	How long have you known your loved one? (Please fill in).			
	Translation	How long have you known your loved one? (Please complete).			
	Committee Outcome	There is how long have you known your loved one? (Please complete).	1.00	1.00	1.00
14	Original	We would like to get feedback from you on how burdensome it was to complete this questionnaire. This information will help guide us in future research. Overall, how much of a burden on you was this questionnaire? (Circle one number).			
	Translation	Please tell us how hard it was to complete this questionnaire. This information will help us to direct our future research. Overall, how laborious was this questionnaire for you? (Circle a number).			
	Committee Outcome	Please tell us How hard was it for you to complete this questionnaire? This information will help us to direct our future research. Considering 0 (zero) as not at all laborious, and 10 (ten) as very laborious. (Circle a number).	1.00	1.00	1.00

* Content Validity Index.

## Data Availability

The data that support the findings of this study are available on request from the corresponding author. The data are not public available due to restrictions e.g., their containing information that compromise the privacy of research participants. That all listed authors meet the authorship criteria and that all authors are in agreement with the content of the manuscript.

## References

[1] Gellie A., Mills A., Levinson M., Stephenson G., Flynn E. (2015). Death: A foe to be conquered? Questioning the paradigm. Age Ageing.

[2] Patrick D.L., Engelberg R.A., Curtis J.R. (2001). Evaluating the quality of dying and death. J. Pain Symptom Manag..

[3] Brooks L.A., Manias E., Nicholson P. (2017). Barriers, enablers and challenges to initiating end-of-life care in an Australian intensive care unit context. Aust. Crit. Care.

[4] Gerritsen R.T., Hofhuis J.G.M., Koopmans M., van der Woude M., Bormans L., Hovingh A., Spronk P.E. (2013). Perception by family members and ICU staff of the quality of dying and death in the ICU: A prospective multicenter study in The Netherlands. Chest.

[5] Mularski R., Curtis J.R., Osborne M., Engelberg R.A., Ganzini L. (2004). Agreement among family members in their assessment of the Quality of Dying and Death. J. Pain. Symptom Manag..

[6] Curtis J.R., Patrick D.L., Engelberg R.A., Norris K., Asp C., Byock I. (2002). A measure of the quality of dying and death. Initial validation using after-death interviews with family members. J. Pain Symptom Manag..

[7] Gerritsen R.T., Koopmans M., Hofhuis J.G.M., Curtis J.R., Jensen H.I., Zijlstra J.G., Engelberg R.A., Spronk P.E. (2017). Comparing Quality of Dying and Death Perceived by Family Members and Nurses for Patients Dying in US and Dutch ICUs. Chest.

[8] Fortes C.P.D.D., Araújo A.P.Q.C. (2019). Check list para tradução e Adaptação Transcultural de questionários em saúde. Cad. Saúde Colet.

[9] Curtis J.R., Downey L., Engelberg R.A. (2013). The quality of dying and death: Is it ready for use as an outcome measure?. Chest.

[10] Downey L., Curtis J.R., Lafferty W.E., Herting J.R., Engelberg R.A. (2010). The Quality of Dying and Death Questionnaire (QODD): Empirical domains and theoretical perspectives. J. Pain Symptom Manag..

[11] Fink-Samnick E. (2016). The Evolution of End-of-Life Care: Ethical Implications for Case Management. Prof. Case Manag..

[12] Pérez-Cruz P.E., Perez O.P., Bonati P., Parisi O.T., Satt L.T., Otaiza M.G., Yáñez D.C., Morgado A.M. (2017). Validation of the Spanish Version of the Quality of Dying and Death Questionnaire (QODD-ESP) in a Home-Based Cancer Palliative Care Program and Development of the QODD-ESP-12. J. Pain Symptom Manag..

[13] Sánchez D.G., Cuesta-Vargas A.I. (2018). Cross-cultural adaptation and psychometric testing of the Quality of Dying and Death Questionnaire for the Spanish population. Eur. J. Oncol. Nurs..

[14] Heckel M., Bussmann S., Stiel S., Weber M., Ostgathe C. (2015). Validation of the German version of the Quality of Dying and Death Questionnaire for Informal Caregivers (QODD-D-Ang). J. Pain Symptom Manag..

[15] Han X., Mei X., Zhang J., Zhang T., Yin A., Qiu F., Liu M. (2021). Validation of the Chinese Version of the Quality of Dying and Death Questionnaire for Family Members of ICU Patients. J. Pain Symptom Manag..

[16] Moslemi M., Nikfarid L., Nourian M., Nasiri M., Rezayi F. (2020). Translation, Cultural, and Age-Related Adaptation and Psychometric Properties of Persian Version of “Quality of Dying and Death” in Nurses Working in Neonatal Intensive Care Units. Indian J. Palliat. Care.

[17] Glavan B.J., Engelberg R.A., Downey L., Curtis J.R. (2008). Using the medical record to evaluate the quality of end-of-life care in the intensive care unit. Crit. Care Med..

[18] Beaton D.E., Bombardier C., Guillemin F., Ferraz M.B. (2000). Guidelines for the process of cross-cultural adaptation of self-report measures. Spine.

[19] Newman I., Lim J., Pineda F. (2013). Content Validity Using a Mixed Methods Approach: Its Application and Development Through the Use of a Table of Specifications Methodology. J. Mix. Methods Res..

[20] Polit D.F., Beck C.T. (2006). The content validity index: Are you sure you know what’s being reported? Critique and recommendations. Res. Nurs. Health.

[21] Zamanzadeh V., Ghahramanian A., Rassouli M., Abbaszadeh A., Alavi-Majd H., Nikanfar A.R. (2015). Design and Implementation Content Validity Study: Development of an instrument for measuring Patient-Centered Communication. J. Caring Sci..

[22] Teles L.M.R., Oliveira A.S., Campos F.C., Lima T.M., Costa C.C., Gomes L.F.S., Oriá M.O.B., Damasceno D.K.C. (2014). Development and validating an educational booklet for childbirth companions. Rev. Esc. Enferm. USP.

[23] Ogrinc G., Davies L., Goodman D., Batalden P., Davidoff F., Stevens D. (2016). SQUIRE 2.0 (Standards for QUality Improvement Reporting Excellence): Revised publication guidelines from a detailed consensus process. BMJ Qual. Saf..

[24] Mokkink L.B., Prinsen C.A.C., Bouter L.M., Vet H.C.W., Terwee C.B. (2016). The COnsensus-based Standards for the selection of health Measurement INstruments (COSMIN) and how to select an outcome measurement instrument. Braz. J. Phys. Ther..

[25] Lino C.R.M., Brüggemann O.M., Souza M.L., Barbosa S.F.F., Santos E.K.A. (2017). The cross-cultural adaptation of research instruments, conducted by nurses in brazil: An integrative review. Texto Contexto-Enferm..

[26] Buonanno P., Jaiola A., Palumbo C., Spinelli G., Terminiello V., Servillo G. (2017). Italian validation of the Amsterdam Preoperative Anxiety and Information Scale. Minerva Anestesiol..

[27] Machado R.S., Oriá M.O.B., Fernandes M.A., Gouveia M.T.O., Silva G.R.F. (2019). Translation and cultural adaptation of death attitude profile revised (DAP-r) for use in Brazil. Texto Contexto-Enferm..

[28] Valer D.B., Aires M., Fengler F.L., Paskulin L.M.G. (2015). Adaptation and validation of the Caregiver Burden Inventory for use with caregivers of elderly individuals. Rev. Latino-Am. Enferm..

[29] Pirola W.E., Paiva B.S.R., Barroso E.M., Kissane D.W., Serrano C.V.M.P., Paiva C.E. (2017). Translation and cultural adaptation of the Shame and Stigma Scale (SSS) into Portuguese (Brazil) to evaluate patients with head and neck cancer. Braz. J. Otorhinolaryngol..

[30] Santana M.T.E.A., Gómez-Batiste X., Silva L.M.G., Gutiérrez M.G.R. (2020). Cross-cultural adaptation and semantic validation of an instrument to identify palliative requirements in Portuguese. Einstein.

